# Supplementary material for: Serological Conversion through a Second Exposure to Inactivated Foot-and-Mouth Disease Virus Expressing the JC Epitope on the Viral Surface
Source: Vaccines (Basel). 2023 Sep 14;11(9):1487. doi: 10.3390/vaccines11091487 (PMC10537882; doi:10.3390/vaccines11091487)
Supplement: Supplementary file 1 [file vaccines-11-01487-s001.zip › vaccines-2553599-supplementary.pdf]

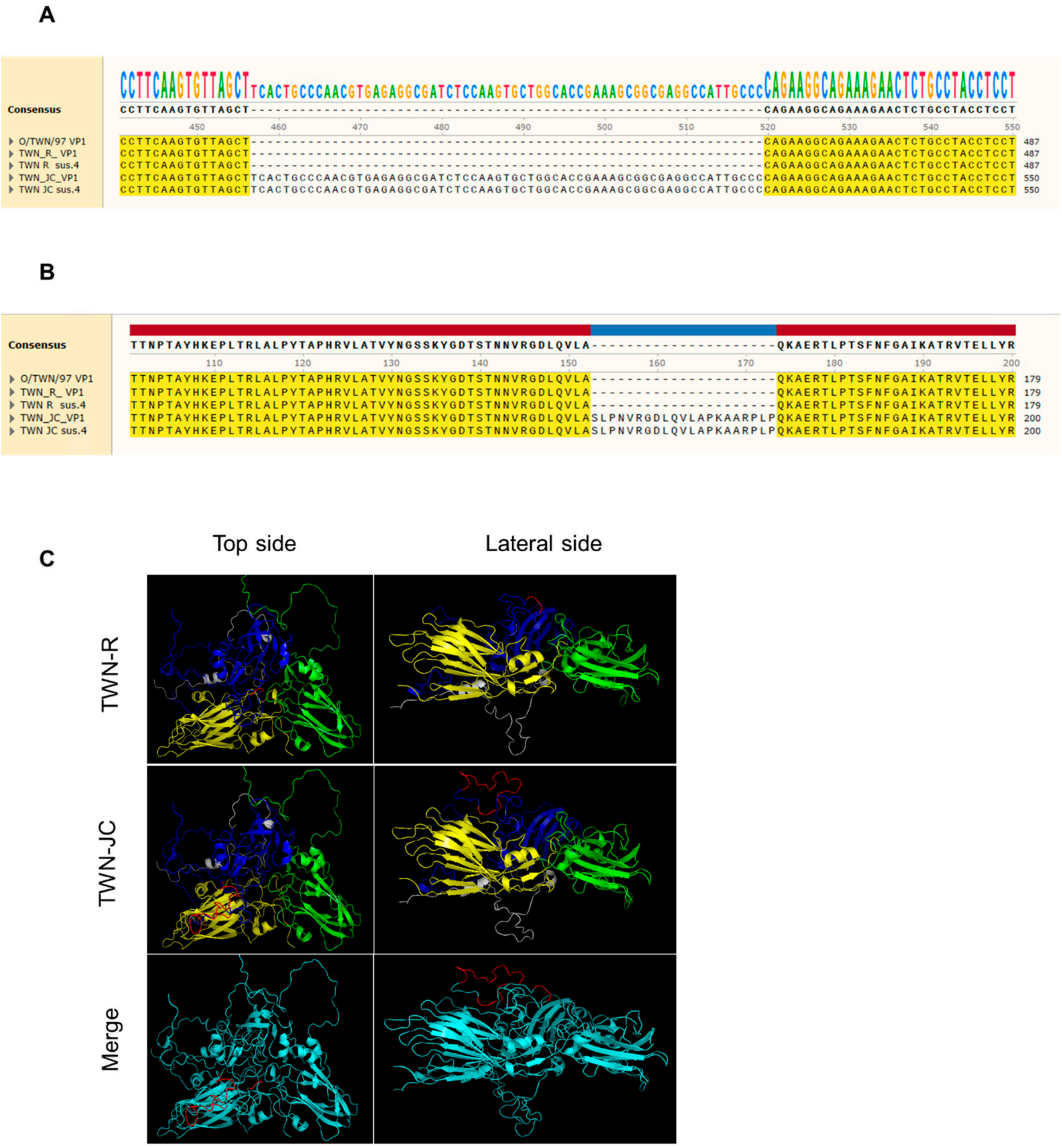

Supplementary Figure S1. Aligned VP1 sequence and modeling of the capsid protomer structure of TWN-R and TWN-JC. Alignment of the TWN-R and TWN-JC nucleotide sequences (A) and amino acid sequences (B) was performed using snap gene software. The sequence encoding the JC epitope was inserted into VP1 between nucleotides 456 and 457 (A) and between amino acids 152 and 153 (B). VP1 sequences of TWN-R and TWN-JC adopted on 4th suspension cell passages were aligned. In each panel, VP1 is shown in blue, VP2 is shown in yellow, VP3 is shown in green, VP4 is shown in gray, and the RGD motif in the GH loop or the linear B-cell epitope is shown in red. Cyan is used to show where the TWN-R and TWN-JC structures overlap.

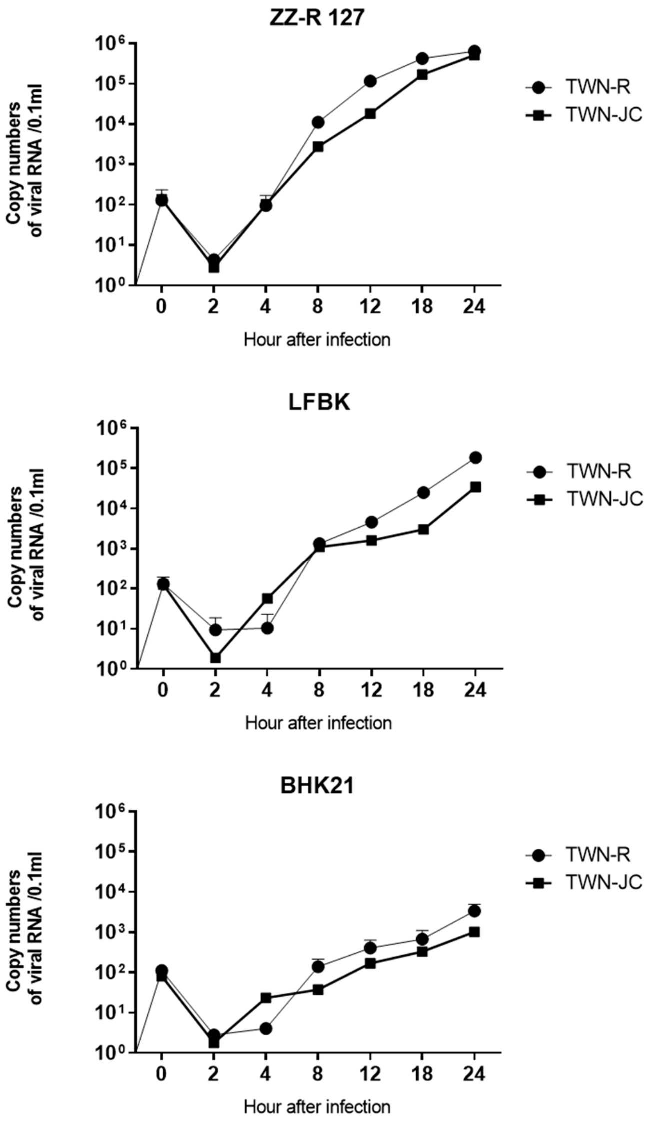

Supplementary Figure S2. Comparison of viral replication between TWN-R and TWN-JC *in vitro*. Three types of cells (ZZ-R 127, LFBK, and BHK-21) were infected at Multiplicity of infection (MOI) of 0.001, and virus growth was measured at 0, 2, 4, 8, 12, 18, and 24 hours post-infection.

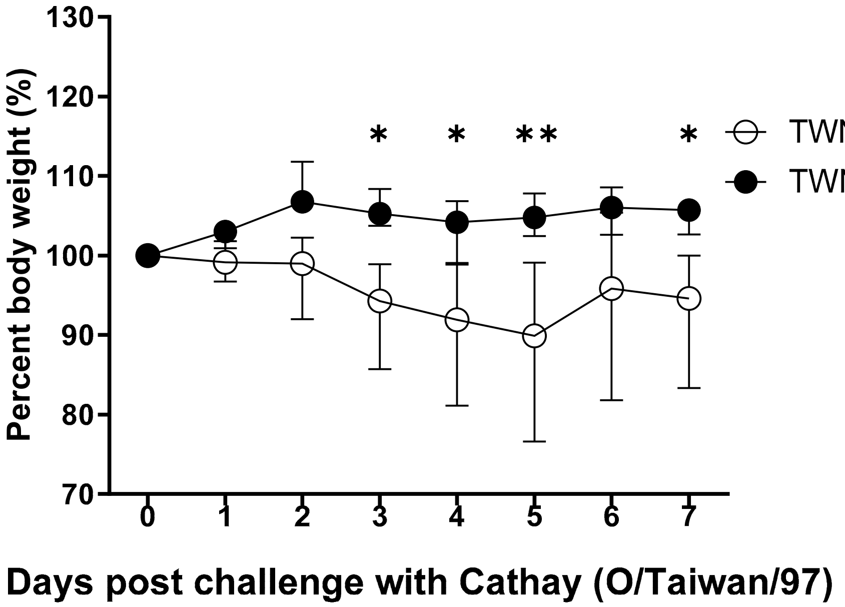

Supplementary Figure S3. Differences in body weight change in mice between TWN-R 1/40 dose group and TWN-JC 1/40 dose group. TWN-R and TWN-JC were vaccinated at 1/40 dose, respectively, and O/TWN/97 virus was inoculated to observe body weight change in mice.
